# A pregnant woman with a surgical site infection after mesh repair of an abdominal wall incisional hernia: a case report

**DOI:** 10.1186/s13256-017-1217-3

**Published:** 2017-03-11

**Authors:** Kana Ozaki, Kenji Tanimura, Yasuhiko Ebina, Kiyonori Kanemitsu, Hideto Yamada

**Affiliations:** 1 Department of Obstetrics and Gynecology, Kobe University Graduate School of Medicine, 7-5-1 Kusunoki-cho, Chuo-ku, Kobe, 650-0017 Japan; 2 Division of Gastrointestinal Surgery, Kobe University Graduate School of Medicine, Kobe, Japan

**Keywords:** Case report, Cesarean section, Incisional hernia, Pregnancy, Surgical mesh, Surgical site infection

## Abstract

**Background:**

Surgical meshes are widely used in incisional hernia repair. However, there are no reports of pregnancies complicated by infection of surgical meshes used for hernia repair. This is the first case report of a pregnant woman who experienced surgical site infection associated with surgical mesh used for repair of an abdominal wall incisional hernia.

**Case presentation:**

We report a case of a 41-year-old pregnant Japanese woman with surgical site infection after mesh repair of an abdominal wall incisional hernia. She was diagnosed with an abdominal wall incisional hernia at 3 months after her third cesarean section, and she underwent an operation of hernia repair with use of monofilament polypropylene mesh 7 months after the third cesarean section. However, a surgical site infection associated with surgical mesh occurred. During antibiotic treatment, she was found to be pregnant. She was referred to our hospital at 13 weeks and 2 days of gestation. The surgeons removed the infected mesh at 16 weeks and 3 days of gestation. Neither the hernia nor infection at the surgical site recurred throughout pregnancy. We planned a cesarean section using a transverse uterine fundal incision method with an upper abdominal incision. The patient delivered a 2478-g healthy female infant.

**Conclusions:**

The present report shows that removal of mesh can safely control surgical site infection during pregnancy.

## Background

Abdominal wall incisional hernia is one of the most common complications of laparotomy, including cesarean section. The rate of patients who need incisional hernia repair after a cesarean section was reported to be 0.47% [1]. Surgical meshes are widely used in incisional hernia repair. A previous report showed that women who received incisional hernia repair with use of surgical mesh before pregnancy successfully had a full-term vaginal delivery [2]. However, there are no reports of pregnancies complicated by infection of surgical meshes used for hernia repair. To the best of our knowledge, this is the first case report of a pregnant woman who experienced surgical site infection associated with surgical mesh used for repair of an abdominal wall incisional hernia.

## Case presentation

**Fig. 1:**
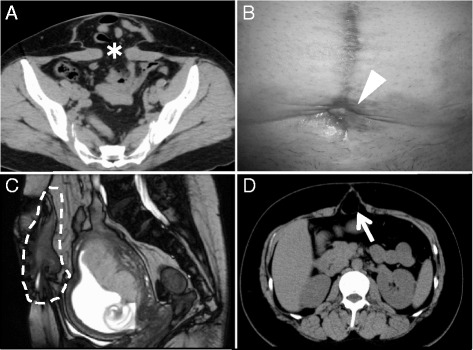
**a** Lower abdominal computed tomographic scan taken 3 months after the third cesarean section. The *asterisk* indicates abdominal incisional hernia with a 10-cm diameter of the hernia orifice. **b** Pus was discharged from a cutaneous fistula in the lower abdominal wall. This image was taken at 13 weeks of gestation. The *arrowhead* indicates a cutaneous fistula. **c** Sagittal, T2-weighted magnetic resonance image of the pelvis taken at 14 weeks of gestation. The *dashed lines* demarcate accumulation of pus surrounding the surgical mesh. **d**. Upper abdominal computed tomographic scan taken 4 months after the most recent cesarean section. The *arrow* indicates incisional hernia in the upper abdominal wall

A 41-year-old pregnant Japanese woman, gravida 4, para 3, underwent three consecutive cesarean sections. She became aware of abdominal distention 3 months after her third cesarean section, which was performed in a private clinic. Therefore, she visited the general hospital, and she was diagnosed as having an abdominal wall incisional hernia (Fig. 1a). She underwent an operation of hernia repair with use of monofilament polypropylene mesh 7 months after her third cesarean section. However, a surgical site infection associated with the mesh occurred 2 months after the hernia repair. Although she received intravenous infusions of antibiotics for 14 days, pus continued to discharge from a cutaneous fistula. She was found to be pregnant at that time.

The patient and her spouse did not desire termination of the pregnancy. She was referred to Kobe University Hospital at 13 weeks and 2 days of gestation, at which time she was found not to have fever, abdominal pain, or peritoneal irritation signs. Her body mass index prior to pregnancy was 24.4 kg/m^2^. Her white blood cell count (8800/μl) and serum C-reactive protein level (0.87 mg/dl) were slightly increased. A cutaneous fistula was present in the lower abdominal wall, and pus discharged from the fistula (Fig. 1b). *Streptococcus agalactiae* and *Peptostreptococcus micros* were isolated by bacterial culture of pus. Magnetic resonance imaging performed at 14 weeks of gestation clearly demonstrated an accumulation of pus surrounding the surgical mesh (Fig. 1c).

Surgeons performed an operation to remove the infected mesh at 16 weeks and 3 days of gestation. During the surgery, it was found that an infection had spread to the soft tissue surrounding the mesh and that the infected tissue strongly adhered to the bladder, omentum, and small intestine. The bladder wall and the serosa of the small intestine developed defects during the adhesiolysis, resulting in a 4-cm, full-thickness opening in the dome of the bladder and a defect of the serosa of the small intestine with a 2-cm width. The bladder mucosa was repaired by using interrupted sutures with 0.2-mm polydioxanone monofilament sutures, and the muscular layer and the serosa of the bladder was repaired by using a continuous suture with 0.2-mm multifilament absorbable sutures. The defect of serosa of the small intestine was repaired by interrupted sutures with 0.2-mm multifilament absorbable sutures. An interrupted one-layer mass closure with 0.35-mm polydioxanone monofilament sutures was used on the peritoneum and abdominal fasciae, and a vertical mattress suture with 0.2-mm nylon sutures was used for skin closure.

The patient received intravenous infusions of ampicillin/sulbactam (12 g/day) and ritodrine hydrochloride for prophylaxis of uterine contraction for 2 weeks. After this operation, the pus discharge completely stopped. The patient was discharged from the hospital 32 days after the operation, when her white blood cell count and serum C-reactive protein level had returned to normal without a uterine contraction or a fever. Neither hernia nor infection at a surgical site recurred during the rest of her pregnancy as well as during the puerperal period.

A cesarean section with an upper abdominal incision, followed by a transverse uterine fundal incision, was planned with the patient’s informed consent. A female newborn weighting 2478 g with Apgar scores of 9 (1 minute) and 9 (5 minutes) was delivered successfully by cesarean section at 37 weeks and 3 days of gestation. The same procedure used in the previous operation to remove the infected mesh was performed for the upper abdominal wall closure. The patient’s blood loss volume was 820 ml. The patient and her child were discharged 8 days later, and the remainder of the hospital course was uneventful. However, an incisional hernia of the upper abdominal wall occurred 4 months after this cesarean section (Fig. 1d). Seven months after the cesarean section, surgeons repaired this hernia by open surgery without use of surgical mesh and by the same procedure for abdominal wall closure used in the previous operations. The incisional hernia of the abdominal wall did not recur for at least 10 months thereafter.

## Discussion

Incisional hernia is one of the many complications of laparotomies, including gynecological surgery and cesarean section. An incisional hernia or strangulation of the uterus is one of the severest complications of incisional hernia during pregnancy. These complications can cause miscarriage, preterm delivery, and intrauterine fetal death [3–7]. In general, pregnant women who have a complication of incisional hernia receive conservative management during pregnancy and undergo an operation of hernia repair after delivery [8]. Recently, repair of incisional hernia with use of surgical mesh has been widely performed. The recurrence rate of hernia after repair without mesh is higher than that of repair with mesh, but the complication rate is similar with both procedures [9]. Surgical site infection associated with surgical mesh is one of the major complications of hernia repair. This condition may be treated by surgical debridement, wound irrigation, mesh removal, and administration of antibiotics [10].

To the best of our knowledge, this is the first case report of a pregnant woman who experienced surgical site infection associated with mesh used for hernia repair. The infected mesh was removed in the second trimester. The pregnancy course was uneventful after the operation, and a healthy newborn was successfully delivered by cesarean section in which an upper abdominal incision was followed by a transverse uterine fundal incision. The transverse uterine fundal incision in a cesarean section is often used to reduce intraoperative blood loss for patients with an adherent placenta complicated by placenta previa [11]. In our patient, there was no adhesion between the upper abdominal wall and the surrounding organs, and the cesarean section was successfully completed. If a lower abdominal incision had been performed, risks of recurrence of abdominal wall incisional hernia and damage to surrounding organs adherent to the abdominal wall would have increased. However, an upper abdominal wall incisional hernia occurred 4 months after the cesarean section. Incisional hernia is likely to occur more frequently in cases with an upper abdominal incision than in those with a lower abdominal incision [12].

## Conclusions

This case report shows that removal of mesh can safely control surgical site infection during pregnancy. It provides useful information for clinical practitioners in the field of perinatal medicine as well as surgery.
